# Cell-derived biomimetic nanomedicines: Emerging theranostic platforms for precision management of atherosclerosis

**DOI:** 10.7150/thno.133733

**Published:** 2026-09-09

**Authors:** Xingji Liu, Zhiyue Wang, Jiaqi Lu, Jie Sheng, Longjiang Zhang

**Affiliations:** 1 Department of Radiology, Jinling Hospital, The First School of Clinical Medicine, Southern Medical University, Nanjing, Jiangsu 210008, China.; 2 Department of Radiology, Jinling Hospital, Affiliated Hospital of Medical School, Nanjing University, Nanjing, Jiangsu 210008, China.; 3 Department of Radiology, Jinling Clinical Medical College, Nanjing University of Chinese Medicine, Nanjing, Jiangsu 210008, China.

**Keywords:** atherosclerosis, biomimetic nanoparticles, drug delivery systems, clinical translation, theranostics

## Abstract

Atherosclerosis (AS), driven by complex cellular interactions, remains a leading cause of mortality worldwide. Conventional therapies often lack diseased blood vessels specificity, limiting therapy efficacy while increasing off-target toxicity. Boasting the biological advantages of cell-derived camouflages, these biomimetic nanomedicines outperform traditional drugs in intraplaque accumulation, circulation half-life and anti-AS efficacy. Current cell-derived biomimetic nanomedicines include whole-cell carriers, cell membrane-coated nanoparticles (NPs), and extracellular vesicles (EVs), all exploitable as novel platforms for plaque imaging and treatment. This review introduces the cellular mechanisms of AS pathology, examines recent advances in biomimetic strategies for AS theranostics and discusses the major barriers in clinical translation including biomaterial design, manufacturing consistency, safety evaluation, and large-scale production.

## Introduction

Coronary artery disease (CAD) remains a primary cause of morbidity and mortality worldwide 1-5. Currently, CAD management has shifted from handling ischemic complications to detecting and treating atherosclerotic plaques before the occurrence of clinical events. Earlier intervention is of vital importance to reduce the global burden of cardiovascular disease 6, 7.

Atherosclerosis (AS), the underlying pathology of CAD, is a chronic inflammatory disease. It involves endothelial dysfunction, lipid accumulation, and fibrous cap development within the arterial wall 8, 9. Current techniques for plaque diagnosis include computed tomography (CT) angiography, magnetic resonance imaging (MRI), and intravascular imaging such as intravascular ultrasound, optical coherence tomography and near-infrared spectroscopy; nevertheless, none is able to directly reveal plaque phenotype and underlying mechanism 10. As a result, they have limited sensitivity for detecting early-stage disease 11-16.

Lipid-lowering therapy, along with surgical or catheter-based revascularization, remains the conventional intervention of AS. Statins and proprotein convertase subtilisin/kexin type 9 (PCSK9) inhibitors lower low-density lipoprotein (LDL) cholesterol. Although these drugs significantly reduce cardiovascular risk, they exhibit limited efficiency in the plaque-targeted delivery, leading to insufficient drug accumulation at lesions and potential adverse effects, such as statin-associated myopathy 17-19. Furthermore, surgical interventions such as coronary stenting and coronary artery bypass grafting can achieve revascularization but are associated with restenosis and graft occlusion 20-22. Given these drawbacks, precise approaches for AS management are urgently required.

Nanomedicine can improve the precision of AS diagnosis and treatment 23-25. Engineered nanoparticles (NPs) can not only deliver drugs in a controlled manner, but also achieve molecular imaging by targeting specific receptors 26. However, one of the main challenges in the clinical translation is the immunological clearance of NPs from the circulation by the reticuloendothelial system. This rapid clearance reduces the blood circulation time of NPs, thereby limiting sustained drug delivery and compromising therapeutic efficacy 27-29. To address this challenge, researchers have incorporated natural cellular delivery systems into nanoparticle designs.

The usage of natural cellular components endows cell-derived biomimetic NPs with extraordinary targeting efficacy to the lesion sites 30, 31. The preserved membrane proteins and surface molecules provide endogenous recognition signals, such as CD47-mediated “don't eat me” signaling and receptor-mediated targeting interactions, which enhance immune evasion and promote accumulation in diseased tissues 32, 33. In general, these NPs can be divided into three categories: whole-cell carriers, cell membrane-coated nanoparticles (CM-CNPs), and extracellular vesicles (EVs). Whole-cell carriers conjugate drugs directly to cells in a bid to improve homing ability and half-life in circulation. CM-CNPs combine biomedical materials, such as metal NPs, nanogels, and metal-organic frameworks, with natural cell membranes obtained from various cell types. These nanoplatforms retain functions such as immune evasion and tissue recognition. EVs, including exosomes and microvesicles, transport biological cargo such as proteins, regulatory RNAs, and lipids intercellularly. Upon uptake by target cells, EVs protect these cargo from endosomal degradation and facilitate their entry into the cytoplasm, enabling precise regulation of cellular functions 34, 35. Collectively, these biomimetic NPs exhibit enhanced delivery and regulatory capabilities, thereby offering a promising avenue to address unmet needs in AS management.

This review explores cell-derived biomimetic nanomedicines for the diagnosis and treatment of AS. We first describe the pathological mechanisms and cellular processes that drive AS progression, then compare whole-cell carriers, CM-CNPs, and EVs and evaluate their applications in plaque imaging and targeted therapy. Moreover, we discuss the potential of clinical translation and the directions for future research.

## Pathogenesis of AS

AS pathogenesis involves coordinated cellular and molecular interactions that drive progressive vascular alterations, underscoring the critical roles of diverse cell populations 36, 37. In this section, we summarize the key cell types involved in AS and their contributions to disease development.

### Endothelial cells (ECs)

Endothelial dysfunction is an early hallmark of AS 38. Atherogenic risk factors, such as disturbed blood flow and oxidative stress, induce endothelial cells (ECs) activation and dysfunction. Activated ECs secrete chemokines, including monocyte chemoattractant protein-1 (MCP-1) and interleukin-8 (IL-8). They also upregulate the expression of vascular cell adhesion molecule-1 (VCAM-1) and intercellular adhesion molecule-1 (ICAM-1), thereby promoting the recruitment and adhesion of circulating immune cells to the vessel wall 9.

Endothelial barrier integrity disruption permits low-density lipoprotein (LDL) to infiltrate the arterial intima. Once retained in the intima, LDL accumulates and undergoes oxidative modification by reactive oxygen species (ROS), forming oxidized LDL (ox-LDL). Ox-LDL further stimulates endothelium and promotes the release of pro-inflammatory mediators, thereby sustaining local inflammation and promoting lesion formation 39-41.

### Monocytes and macrophages

Monocytes are key components of the innate immune system and serve as the major contributors of macrophages within plaques 42. Circulating monocytes are recruited to the activated ECs through coordinated chemokine and adhesion-molecule signals. Monocyte chemoattractant protein-1 (MCP-1) binding to C-C chemokine receptor type 2 (CCR2) drives monocyte chemotaxis, whereas endothelial vascular cell adhesion molecule-1 (VCAM-1) and intercellular adhesion molecule-1 (ICAM-1) mediate firm adhesion. Recruited monocytes then transmigrate across the endothelial layer into the intima, where they differentiate into macrophages and contribute to plaque formation 43.

Following transmigration and differentiation, macrophages internalize oxidized low-density lipoprotein (ox-LDL). Continued lipid uptake transforms the macrophages into foam cells, which is a typical feature of atherosclerotic lesions. Foam cells secrete pro-inflammatory cytokines, including tumor necrosis factor-alpha (TNF-α) and interleukin-1 beta (IL-1β), which increase the endothelial adhesion molecules and enhance the chemokine production 44, thereby amplifying monocyte recruitment and sustaining a feed-forward inflammatory loop 45-47.

### Platelets (PLTs)

In response to vascular injury, PLTs adhere to exposed subendothelial collagen, initiating hemostasis and amplifying vascular inflammation 48. This initial adhesion is mediated by glycoprotein VI (GPVI) and integrin α_2_β_1_ (GPIa/IIa). Subsequent platelet activation induces a conformational change in integrin αII_b_β_3_ (GPIIb/IIIa), enabling fibrinogen to cross-link adjacent PLTs. This interaction promotes PLTs aggregation and stabilizes PLTs adhesion to the injured vessel wall.

Once activated, PLTs translocate P-selectin from α-granules to the cell surface, where it binds to P-selectin glycoprotein ligand-1 (PSGL-1), a high-affinity receptor on circulating monocytes. Through this interaction, activated PLTs support monocyte adhesion and recruitment to atherosclerotic plaques 49-53.

### Neutrophils

Neutrophil recruitment to atherosclerotic lesions is driven partly by interleukin-8 (IL-8)-CXCR1/CXCR2 signaling. Once activated, neutrophils produce ROS and release neutrophil elastase, thereby exacerbating endothelial dysfunction and vascular inflammation. Furthermore, neutrophils amplify vascular inflammation through the release of neutrophil extracellular traps (NETs), which activate macrophages and dendritic cells, enhance pro-inflammatory cytokine production and accelerate plaque progression 54-57.

### Vascular smooth muscle cells (VSMCs)

VSMCs play both structural and regulatory roles during the development of AS 58. As plaques develop, VSMCs migrate from the media to the intima, where they produce collagen and extracellular matrix (ECM) components. These components help preserve fibrous cap integrity and contribute to plaque stability.

However, inflammatory signals and oxidative stress can induce VSMC phenotypic switching from contractile state to synthetic state. Synthetic VSMCs lose their contractile function and secrete matrix-degrading enzymes, such as matrix metalloproteinases (MMPs), as well as pro-calcific factors. These secreted factors degrade the ECM, thereby weakening the fibrous cap and increasing plaque vulnerability to rupture 59, 60.

### Red blood cells (RBCs)

RBCs contribute to atherosclerotic plaque progression primarily in the context of intraplaque hemorrhage (IPH). Intraplaque hypoxia and oxidative stress promote the formation of neovessels within atherosclerotic lesions. However, these are often immature, fragile and highly permeable, making them prone to rupture and facilitating RBC extravasation into the plaque.

Following IPH, extravasated RBCs undergo hemolysis and release damage-associated molecular patterns (DAMPs), thereby promoting necrotic core expansion, amplifying local inflammation, and increasing plaque vulnerability 61, 62.

### Summary

AS is a chronic and complex disease that develops over several decades 63, 64. Multiple vascular and immune cells play distinct but interconnected roles at different stages of disease. A better understanding of these cellular mechanisms may facilitate the rational design of biomimetic nanomedicines and enable the identification of promising cellular targets for the diagnosis and treatment of AS.

## Advances in cell-derived nanotechnology for the theranostics of AS

According to the specific functions of different cells mentioned before, various cell-derived NPs have been developed for the treatment of AS 65. These NPs retain native cellular functions and modes of intercellular communication, thus achieving improved immune evasion, prolonged circulation, and enhanced plaque accumulation 66-68. The following section summarizes the major types of cell-derived nanomedicines (**Tables 1 and 2**) and introduces their benefits and limitations in AS management.

### Whole-cell-based vehicles

Whole-cell vehicles use intact cells to carry therapeutic agents while preserving their cellular structure and function. Depending on the source cell, these carriers can evade immune clearance, prolong circulation, and migrate toward inflamed tissues. Therefore, these nanomedicines can increase drug accumulation in atherosclerotic plaques and improve therapeutic efficacy 69, 70. Their prolonged circulation and inflammation-directed homing make whole-cell carriers suitable for targeting intermediate and advanced plaques with higher inflammatory activity 71. Meanwhile, anti-AS agents encapsulated within cells usually have improved biocompatibility, which is another merit of whole-cell-based vehicles 72. The intact cell structure shields the therapeutic cargo from immune recognition and complement activation, supporting the use of whole-cell carriers in AS imaging and treatment.

RBC-based vehicles are among the earliest biomimetic drug delivery nanoplatforms. Mature RBCs lack nuclei, facilitating their application as bioengineered carriers 73. Shen et al. developed a RBC-based nanodrug for simvastatin delivery. The RBC structure reduced clearance by the mononuclear phagocyte system and prolonged the circulation half-life by 4.3-fold compared with the free-drug formulation 74.

Immune cells respond to chemotactic signals and migrate toward inflammatory microenvironments. Zeng et al. exploited this property to develop a dual-modality nanoprobe for early AS diagnosis. They used macrophages to encapsulate gold NPs. Owing to the homing ability of macrophages, this probe can be directed to atherosclerotic plaques and enable prolonged real-time monitoring through ultrasound/photoacoustic (US/PA) imaging 75. Similarly, neutrophil-based melanin NPs have also been used in AS therapy. In vivo, these nanodrugs delayed disease progression and reduced plaque area by 24.7%, demonstrating that immune cell-biomimetic nanomedicine can effectively deliver therapeutic medicine to inflamed plaques 76.

### Cell membrane-coated nanoparticles (CM-CNPs)

Although whole-cell carriers offer great biocompatibility and cell-specific targeting, their application is limited by the large-scale production and long-term storage. The need to maintain structural integrity and functional stability during manufacturing and preservation makes it challenging to meet clinical requirements. These constraints led to the development of CM-CNPs, which preserve cellular functions without requiring intact cells 77.

CM-CNPs are fabricated via coating NP cores with natural cell membranes 78. Unlike whole-cell carriers, CM-CNPs can encapsulate hydrophobic agents or cytotoxic drugs within their cores and achieve controlled drug release 79-81. This ability makes them suitable for targeting different stages of AS and obtain both diagnostic and therapeutic purposes. The following sections describe the characteristics and applications of four major types of CM-CNPs in AS.

#### RBCs

RBC is an ideal source for membrane-coated drug delivery. The anucleate structure of mature RBCs simplifies membrane isolation and modification, while the biconcave shape provides a large surface area for molecular exchange 82. Compared with 5 to 90 hours of neutrophils and 5 to 7 days of PLTs in circulation, human RBCs circulate for about 120 days, providing a prolonged circulation time that facilitates sustained drug exposure and enhances drug accumulation at atherosclerotic lesions. RBCs are also cleared minimally by immune cells, making them well suited to targeted drug delivery 83.

In AS, the CD47 protein serves as a “do not eat me” signal that confers immune evasion capability upon RBCs, which extends circulation half-life and enhances targeted delivery to atherosclerotic lesions 84. Based on this property, Liu et al. coated a ROS-responsive fluorescent molecule (CH1055) and a stable fluorescent dye (Cy7) with RBC membranes to construct two biomimetic nanoprobes. The RBC membrane coating prolonged the probe’s in vivo half-life from 1.55 to 2.82 h, enabling dynamic monitoring of inflammatory foci (Figure 2D-F) 85.

Oxidative stress in the local microenvironment is a key pathogenic factor of AS. Synthesizing anti-ROS agents with RBC membranes shows great potential for AS therapy. Karami et al. synthesized nanoghost (NG) by encapsulating glyburide within PLGA NPs coated with RBC membranes. Histochemical analysis revealed that NG reduced foam cell burden by 1.79-folds compared with un-coated NPs, demonstrating the therapeutic benefit of biomimetic encapsulation 86. Similarly, an miR-10a-loaded RBC membrane–coated liposomal nanomedicine showed considerable potential for the targeted treatment of AS. This nanodrug considerably inhibited the progression of AS in vivo. This effect was confirmed by Oil Red O staining of mouse aortic sections, which showed an approximately 45% plaque reduction relative to Atorvastatin treatment controls (Figure 2A-C) 87.

Although RBC membrane–based nanomedicines show considerable promise in AS management, their clinical translation is constrained by low production yields. To address this limitation, Cao et al. developed a self-assembled RBC membrane-based nanodrug for AS management. They introduced the PS-binding peptide CLIKKPF onto NPs, enabling specific interactions with PS on RBC membranes. This method drove spontaneous self-assembly and enabled efficient membrane coating without complex processing methods such as electroporation 88.

In summary, RBC membrane-based biomimetic nanomaterials show great potential for AS theranostics. These nanomaterials retain key membrane proteins, such as CD47, which helps them evade immune clearance. This feature prolongs their circulation time and enhances drug stability. However, the lack of intrinsic active targeting limits their accumulation at plaque sites. Future studies should explore active targeting strategies to improve plaque-specific delivery efficiency 89.

#### Macrophages

Macrophages coordinate innate immune responses through phagocytosis and immune signaling 90. During AS progression, activated endothelial cells (ECs) express high levels of vascular cell adhesion molecule-1 (VCAM-1), which interacts with ligands on macrophage membranes, thereby promoting macrophage homing to plaques. As a result, macrophage membrane coating of NPs can facilitate delivery of imaging agents or therapeutics to plaques 91, 92. For example, Li et al. developed macrophage membrane-coated Prussian blue NPs, termed MM@HGPBRD. In vivo studies showed that MM@HGPBRD produced stronger MRI signal enhancement in inflamed plaques than un-coated NPs (Figure 3F-G) 93. Similarly, Duan et al. developed a macrophage membrane-cloaked fluorescent nanoprobe (LC-MM). The LC-MM nanoprobe exhibited higher fluorescence intensity in plaque regions than un-coated NPs. These results demonstrated its potential for real-time imaging of vascular inflammation (Figure 3A-E) 94.

To achieve therapeutic effects, Cai et al. developed macrophage membrane-biomimetic liposomes (R@MLP) with a retinoic acid loaded core. This biomimetic design enabled the targeting and blocking of apoptotic cells with elevated CD47 levels in atherosclerotic plaques. In vivo studies demonstrated an approximately 70% reduction in plaque area compared to controls (Figure 4A-D) 95. Similarly, Li et al. encapsulated evolocumab, a PCSK9 inhibitor, in macrophage-coated nanoparticles ((Lipo+M)@E NPs). Fluorescence imaging revealed a 1.59-fold higher plaque accumulation of (Lipo+M)@E NPs than un-coated NPs. This enhanced delivery resulted in superior efficacy, as (Lipo+M)@E NPs reduced plaque area to 3.78%, compared with 12.44% in the Lipo@E NP group (Figure 4H) 96.

Foam cells are key contributors to atherosclerotic plaque formation, and their apoptosis promotes plaque instability. Cheng et al. developed macrophage membrane-coated Au-ZnO Schottky junctions (Au-ZnO@MM) to enhance foam cell elimination and thereby inhibit necrotic core formation. In vivo experiments showed an approximately 50% reduction in necrotic core area compared with control NPs, demonstrating the strategy’s potential to mitigate AS progression (Figure 4E-G) 97.

In summary, macrophage membrane-based nanomedicines offer substantial promise for AS therapy as an active targeting strategy 98. Future studies should focus on optimizing macrophage membrane modifications to achieve stable recognition and selective binding to pathological targets, thereby broadening the clinical translation potential of macrophage membrane-based nanoplatforms 99.

#### PLTs

PLTs play essential roles in vivo, including in hemostasis, wound healing, and inflammatory modulation. Compared to nucleated cells, PLTs have fewer surface antigens and lower immunogenicity, making them attractive candidates for biomimetic drug delivery. Leveraging their targeting capabilities toward injured endothelial cells and thrombi in atherosclerotic lesions, PLT membrane–coated NPs (PLT M–CNPs) can selectively accumulate in the sites of vascular injury 51-53, 100. This section reviews the application of PLT membrane-based nanotechnology in AS theranostics.

PLT M–CNPs show substantial potential for AS imaging. Zhou et al. designed a PM@CBNP nanoprobe based on the photosensitizer Ce6 for fluorescence imaging. This nanoprobe showed preferential accumulation in atherosclerotic plaques, resulting in 1.5-fold higher fluorescence intensity than bare micelles at 48 h post-injection 101. To achieve accurate assessment, PM/Fe_3_O_4_@PLGA was developed as an MRI contrast agent for AS diagnosis. In vivo, PM/Fe_3_O_4_@PLGA showed a specific signal increase in the left carotid artery (LCA) at 24 h post-injection, but the un-coated controls gave almost no signal 102.

To enable real-time assessment of plaque progression, photoacoustic nanoprobes have been developed for AS diagnosis 10, 103, 104. For example, Ma et al. constructed PGA@PMP by encapsulating the photoacoustic agent PMeDTDPP-THBTD within a PLT membrane. Carotid photoacoustic tomography revealed that PGA@PMP produced strong and specific signals at plaque sites, enabling real-time visualization of lesion progression 105. Similarly, PLT membranes were used to cloak a near-infrared-II fluorescent dye, enabling real-time plaque imaging, as demonstrated by Chai et al. 106.

During plaque formation, PLTs adhere to the vascular endothelium by binding to exposed fibrinogen through the integrin αII_b_β_3_. Leveraging this targeting capability, Zhang et al. synthesized PBP@siR@PM NPs by encapsulating black phosphorus nanosheets within PLT membranes. The results demonstrated that these biomimetic NPs exhibited 2-fold greater accumulation at plaque sites than un-coated controls (Figure 5A-B) 107. Similarly, PLT membrane-coated empagliflozin-loaded PCL-PEG polymeric micelles showed considerable potential for targeted AS treatment. In vivo, PM@EPPT reduced aortic plaque area by 43% compared with free empagliflozin (Figure 5C-E) 108.

Fontana et al. developed a multilayer-modified nanosystem comprising a curcumin-loaded lignin core coated with tannic acid–iron complexes and an outer layer of PLT membrane fragments. The tannic acid–iron layer increased macrophage uptake from approximately 20% to 30%–40%, while subsequent PLT membrane coating further enhanced uptake to ≥60%. These results demonstrated that membrane modification techniques can enhance cellular targeting and improve plaque-directed drug delivery (Figure 5F-G) 109.

PLT membrane–based nanomedicines offer substantial advantages for AS theranostics, owing to their intrinsic affinity for injured vessels. However, the potential pro-thrombotic risk associated with these nanomaterials remains a key concern for clinical translation. To address this limitation, dynamically reconfigurable nanomaterials have emerged as a promising strategy. Future innovations should focus on smart biointerfaces incorporating stimuli-responsive molecular switches, such as shear stress–activated receptors, to enable self-regulated adhesion to plaques and detachment from healthy endothelium after treatment, thereby improving safety 110-112.

#### Neutrophils

Neutrophils act as frontline components of innate immunity. They respond to infection and tissue injury through phagocytosis, degranulation, and NET formation 113. In early AS, activated neutrophils are recruited to damaged ECs through membrane proteins such as CD11b/CD18. This ability to rapidly respond to inflammatory signals makes neutrophil membrane-coated NPs promising candidates for the diagnosis and treatment of AS 56, 114.

Leveraging this property, Zhang et al. developed neutrophil membrane–based MRI nanoprobes (NNP^ST^), which exhibited a time-dependent reduction in T_2_ signal intensity within atherosclerotic plaques. At 24 h post-injection, NNP^ST^ revealed a 67% signal decrease (Figure 6A-E) 115. To enhance production efficiency, Liu et al. engineered neutrophil-microbubble complexes (Neu-Balloons). These NPs maintained the same fluorescence intensity with a 60% cut-off of the neutrophils needed, thereby potentially addressing payload limitations in atherosclerotic plaque imaging (Figure 6F-H) 116.

Neutrophil M–CNPs exhibit substantial anti-inflammatory potential. Liang et al. designed biomimetic NPs to deliver superoxide dismutase and catalase, thereby promoting macrophage polarization from the pro-inflammatory M1 to the anti-inflammatory M2 phenotypes. Treatment with P5c/S/C increased the proportion of M2 macrophages from 0.9% to 4.6% and reduced M1 macrophage proportion from 27.9% to 2.8% (Figure 7D-E) 117. Similarly, Li et al. developed a neutrophil membrane–fused liposome (PtdSer-NM-Lipo/Fer-1) that achieved a ROS suppression rate of 38.26%, which was substantially higher than the 18.00% suppression observed with free Fer-1 118.

Gene silencing with antisense oligonucleotides (ASOs) is another promising therapeutic strategy for AS. Liu et al. developed neutrophil membrane–camouflaged NPs to deliver an ASO against microRNA-155 to ECs in atherosclerotic lesions. CD18 on the neutrophil membrane bound to ICAM-1 expressed on inflamed endothelium, thereby promoting ASO delivery. The delivered ASO subsequently suppressed the NF-κB pathway, alleviating endothelial inflammation and inhibiting monocyte recruitment. In vivo studies showed that the aortic lesion area decreased from 20% to 6.77%. These results confirmed the high therapeutic efficacy of the treatment (Figure 7A-C) 119.

Neutrophil membrane-coated nanomedicines have attracted increasing attention for AS theranostics because they retain neutrophil-derived chemotactic and adhesive properties, enabling efficient accumulation in inflamed vascular tissues. However, unintended activation by inflammatory signals from non-target tissues may cause off-target accumulation, reduce targeting accuracy, and diminish therapeutic effects, thereby limiting clinical translation 120-122. Therefore, neutrophil membranes require modifications that limit nonspecific activation while preserving inflammation-directed drug delivery.

### Extracellular vesicles (EVs)

EVs mediate intercellular communication by transporting proteins, lipids, nucleic acids, and other bioactive molecules. Based on their size, structure, and origin, EVs can be classified into three types: exosomes, microvesicles, and apoptotic bodies. Among the different EV types, exosomes are particularly useful for biomedical applications because of their nanoscale size of approximately 40 to 200 nm. In particular, mesenchymal stem cell (MSC)-derived exosomes can interact with PLTs and monocytes which are important components of the plaque microenvironment. Moreover, the favorable drug loading efficiency of EVs enables effective drug delivery to atherosclerotic plaques 123-126.

#### EVs as drug carriers

EVs can transport therapeutic cargo to selected cells and tissues 127. Zeng et al. developed a delivery platform by camouflaging ginsenoside Rb1/probucol co-assembled NPs with macrophage-derived microvesicles. The structure could retain adhesion proteins, such as VLA-4 and Mac-1, which enhanced targeting to plaques and reduced off-target effects 128.

#### EVs applied in RNA-based therapeutics

RNA interference (RNAi), including microRNA (miRNA) and small interfering RNA (siRNA), is being investigated as a treatment for AS. Their therapeutic use depends on efficient delivery, because RNA is highly susceptible to degradation in vivo. Thus, RNA cargo needs to reach atherosclerotic lesions, escape endosomal degradation, and enter the cytoplasm to silence its target genes 129, 130. Li et al. developed platelet membrane-fused EVs loaded with miR-21a-5p, termed P-EVs. Inflammatory macrophages internalized the P-EVs through endocytosis. The vesicles then escaped lysosomal degradation and released the miRNA into the cytoplasm. In vitro and in vivo studies showed that this treatment upregulated the cholesterol efflux transporters ABCA1 and ABCG1 and reduced plaque area from 26.15% to 14.96% (Figure 8A-C) 131.

Similarly, neutrophil membrane-engineered EVs (NVEVs) delivering miRNAs such as miR-206-3p have shown promising therapeutic efficacy (Figure 8D) 132. To enhance scalability, Jiang et al. developed synthetic exosome-mimetic nanovesicles coated with PLT membranes (P-ENVs), achieving a 250-fold increase in yield compared to conventional EVs while maintaining therapeutic performance (Figure 8E-F) 133.

#### Challenges and future perspectives

Despite these advances, EV-based therapeutics face critical challenges. EV composition and structure are highly heterogeneous, influenced by the cell type of origin, physiological conditions, and production methods. Consequently, the development of standardized isolation and production protocols is imperative for clinical practice 134, 135.

## Targeting strategies for distinct atherosclerotic plaque phenotypes

Several microenvironment-targeting CM-CNP formulations have yielded therapeutic effects in experimental models. Their targeting performance, however, may be compromised considering that the plaque environment keeps changing throughout AS progression. Membrane source, therapeutic cargo, and release profile should therefore match the disease stage and plaque phenotype. The following sections introduce strategies tailored to specific stages and phenotypes (**Tables 3 and 4**).

### Targeting strategies based on disease stage progression

Atherosclerotic lesions can be divided into early, progressive, advanced, and thrombotic stages. Each stage presents different cellular and molecular targets for biomimetic nanomedicines.

Early AS is characterized by endothelial dysfunction, increased vascular permeability, and mild inflammation 37. In this situation, neutrophil membrane-based biomimetic vehicles have emerged as a reliable option because they can target inflamed endothelium 56, 114. An example is the study by Wei et al. They developed a neutrophil membrane-coated miRNA nanoplatform to restore endothelial function and alleviate plaque progression 132.

As plaques progress, lipid deposition increases and inflammation exacerbates 46. During this stage, immune cells actively migrate toward atherosclerotic lesions. Immune cell-mimetic NPs can use this property to head to diseased sites. (Lipo+M)@E NPs were developed on this basis for AS management. They used the natural homing ability of macrophages to target and treat atherosclerotic plaques 96.

Advanced plaques contain necrotic cores and undergo extensive extracellular matrix remodeling 136. Oxidative stress also becomes a dominant feature of the plaque microenvironment. Stimuli-responsive biomimetic systems, particularly ROS-responsive systems, are therefore being explored for AS management 137. Liu et al. developed macrophage membrane-coated nanomedicines with a ROS-sensitive agent. Oxidative stress in atherosclerotic plaques triggers drug release, and macrophage membrane coating helps them evade immune clearance and accumulate at lesion sites 138.

Plaque rupture and subsequent thrombosis are the most dangerous stage. These thrombi may dislodge and obstruct blood vessels and trigger acute cardiovascular events, necessitating precise delivery of thrombolytic agents. PLT membrane-based nanocarriers are well suited for this task because of their naturally homing to thrombotic sites 139. Jiang et al. developed PSr@PT NPs that use PLT membrane-mediated adhesion to thrombi. As a result, the NPs can reduce oxidative injury and disrupt pathogenic interactions between PLTs and the endothelium 140.

### Targeting strategies based on plaque phenotype

Clinicians generally classify plaques as stable or vulnerable according to their structure and risk of rupture. Because vulnerable plaque rupture causes many acute cardiovascular events, most biomimetic nanomedicine studies target this phenotype 141.

Vulnerable plaques, characterized by thin fibrous caps, large necrotic cores, and dense immune cell infiltrates, are unstable and prone to rupture. Activated macrophages release pro-inflammatory mediators and matrix metalloproteinases (MMPs), which degrade the extracellular matrix (ECM) and weaken the fibrous cap 18, 142-144. Therefore, the treatment strategy in this case focuses on suppressing inflammation, limiting ECM degradation and stabilizing the plaque 145. Macrophage and neutrophil membrane-coated nanomedicines can use inflammation-associated adhesion and chemotactic signals to reach inflamed endothelium and necrotic regions. Furthermore, some formulations function as biological decoys, and they can bind to pro-inflammatory cytokines and disrupt inflammatory pathways 146.

Stable plaques have thicker fibrous caps and a lower risk of rupture 64. Treatment aims to preserve vascular homeostasis and prevent transition to a vulnerable phenotype. However, weak inflammatory signals and low vascular permeability will restrict drug accumulation in these lesions. Under this condition, the extended circulation of RBC membrane-coated NPs and EVs increases drug accumulation in plaque, thereby supporting sustained treatment against plaque progression 83.

### Connecting pathology with biomimetic design

In summary, disease stage reflects the temporal progression of AS, while plaque phenotype exhibits structural stability and rupture risk. Considering both sides can guide the rational design of CM-CNPs and support more precise and effective treatment strategies for AS.

## Imaging-guided and feedback-regulated theranostic strategies

Studies often evaluate the diagnostic and therapeutic functions of biomimetic nanomedicines separately. Theranostic platforms integrate both functions, allowing clinicians to track drug delivery and assess treatment response.

Imaging-guided therapy combines therapeutic cargo with diverse probes in the same nanoparticle. The imaging signal reveals NPs distribution and intraplaque accumulation, providing information that can guide treatment decisions. Macrophage membrane-coated NPs, for example, utilize the chemotactic properties of macrophages to reach atherosclerotic lesions. Incorporating a fluorescent probe into these carriers allows plaque localization and therapeutic response to be assessed at the same time 138.

Feedback-regulated platforms add stimulus-responsive drug release to imaging-guided therapy. Xu et al. developed an ultrasound-responsive nanoplatform in which ultrasound triggered therapeutic cargo release, while feedback signals reported treatment response. These functions allowed dynamic adjustments of treatment strategies according to the response 147. Future research should focus on standardized biomimetic platforms that integrate imaging, drug delivery, and stimuli-responsive release. Combining these functions may further improve the accuracy and efficiency of AS treatment.

## Clinical translation of biomimetic nanomedicines

Key steps of clinical translation include target validation, formulation optimization, preclinical evaluation of efficacy and safety, and phased clinical trials. Data on pharmacokinetics and manufacturing quality are also required 148, 149.

Cell-derived nanomedicines have shown great therapeutic effects in preclinical AS models, but their clinical development remains limited. So far, no cell-derived nanomedicine has entered a clinical trial specifically in AS treatment 150. Nevertheless, experience from other disease indications may guide AS nanomedicine development. ERYtech Pharma developed eryaspase, which encapsulates L-asparaginase within RBCs as an adjunct to chemotherapy. Trials in acute lymphoblastic leukemia and pancreatic cancer has been preliminarily demonstrated safety and therapeutic activity 151, 152. Similarly, Direct Biologics developed ExoFlo, an extracellular vesicle product derived from bone marrow mesenchymal stem cells, for patients with acute respiratory distress syndrome 153.

For AS, the growing number of patent applications for biomimetic NPs reflects industrial interest and translational potential (**Table 5**), which offers a potential platform for disease treatment.

Future trials should include imaging endpoints that measure plaque burden, composition, and inflammatory activity. Regulatory assessments must also address the membrane source and batch-to-batch consistency of each formulation. Standardized characterization methods and good manufacturing practice (GMP)-compliant production shall produce reliable clinical-grade materials.

## Challenges and future directions

### Overcoming translational hurdles

One of the primary issues is the gap between laboratory design and clinical application. Due to insufficient studies on clinical translation of cell-derived NPs, the potential side effects of these endogenous carriers are still unclear.

Large-scale production is another problem. Critical procedures such as mechanical extrusion and sonication must be optimized in order to satisfy clinical standards and meet clinical demands. In addition, most studies have examined only short-term biological safety, while the long-term safety and potential immune reaction remain insufficiently evaluated 154, 155.

### Optimizing material design

Material design of AS-targeted nanomedicine needs both parameter optimization and upgraded function. PLT-mimetic NPs, for example, can capture circulating procoagulant factors, which will reduce thrombosis and support vascular repair. Meanwhile, macrophage-mimetic NPs retain membrane-associated pathogen-recognition functions. Studies of macrophage membranes derived from Porphyromonas gingivalis (Pg)-treated cells suggest that MM/STNPs can eliminate Pg and suppress inflammation at infected sites 136, 156, 157.

Besides cell membrane coating, surface modification by organelle-derived membranes, including mitochondrial and lysosomal membranes, can use homotypic interactions to direct therapeutic cargo toward specific organelles. Also, organelle-specific proteins and bioactive components may regulate cellular signaling, adding therapeutic activity at intracellular and intercellular levels 158-160. Zeng et al. developed an endoplasmic reticulum membrane-based mRNA nanovaccine (P-(H)-m@EMLN), in which endoplasmic reticulum membrane components enhanced endosomal escape and immune activation 161. Such strategies for modulating intracellular trafficking pathways may provide new opportunities for designing multifunctional nanoplatforms for AS therapy.

Extracellular matrix (ECM)-inspired nanomedicines represent another new direction of biomimetic material design. ECM maintains vascular architecture and homeostasis by supporting cells and regulating the vessel microenvironment 162, 163. Current ECM-mimetic materials include nanofibrous scaffolds composed of hyaluronic acid or silk fibroin. Li et al. developed ChS-PDANP@ADSCs, which is a hydrogel utilizing chondroitin sulfate (ChS), a major glycosaminoglycan in the ECM, to promote vascular regeneration and tissue repair 164.

### Clinical assessment

Murine models, especially *ApoE^-/-^* and *LDLR^-/-^* mice, dominate the animal studies in current AS research because of their low cost and well-characterized genetics. These models can develop atherosclerotic lesions but rarely reproduce spontaneous plaque rupture or advanced vulnerable plaques observed in humans 165.

Large-animal models, to a certain degree, can simulate other aspects of the pathology of human AS. Swine models have comparable coronary anatomy and hemodynamics; Non-human primates can develop advanced lesions with features such as intraplaque hemorrhage. Nevertheless, these models involve higher costs and raise greater ethical and procedural concerns 166.

In addition, humanized models and organoid platforms can reproduce human pathological conditions more accurately. Blood vessel organoids (BVOs) derived from induced pluripotent stem cells are providing new approaches for assessing drug activity and safety under pathological conditions. Co-culturing BVOs with monocytes and exposing them to electronegative low-density lipoprotein (eLDL) and TNF-α recreates key inflammatory features of the atherosclerotic microenvironment. This system allows measurements of nanodrug uptake, therapeutic activity, and toxicity in human-derived vascular tissue and may improve the prediction of clinical responses 154, 167, 168.

### AI-driven nanoplatforms

Artificial intelligence (AI) offers opportunities to design personalized nanomedicines for AS. AI algorithms can analyze plaque heterogeneity and help identify nanocarrier designs that better match the patients. They can also predict how NP properties, including composition, size, and surface modifications, affect biological interactions 169, 170. Chen et al. used a machine learning-assisted quantitative structure-activity relationship (QSAR) model to screen anti-atherosclerotic polyphenols from *Salvia miltiorrhiza* and *Carthamus tinctorius*. The team assembled the selected compounds into a metal-phenolic network termed SSPH-MPN. In vivo, SSPH-MPN reduced the plaque burden by 30% compared to the model group 171. Similarly, Shirokii et al. developed a LightGBM model to predict the cytotoxicity of inorganic nanomaterials in different cell types. Compared with previous ML models, LightGBM enabled rapid, interpretable, and quantitative prediction of concentration-dependent cytotoxicity across diverse nanomaterials and cell lines. This approach provides experimental scientists an opportunity to perform primary *in silico* candidate screening and reduces the need for labor-intensive experiments 172.

## Conclusion

Cell-derived biomimetic platforms exploit the advantages of whole cells, cell membranes and EVs, thus increasing drug delivery to atherosclerotic lesions, prolonging half-life in circulation and ultimately, enhancing anti-AS efficacy. Several problems remain though, particularly in large-scale production, standardization, and clinical translation. To address these problems, future research should concentrate on optimizing nanoplatform design and refining the membrane-coating processes to enable the industrial-scale production of CNPs. Meanwhile, establishing standardized quality control systems, including the assessment of membrane integrity and functional stability, will be critical for ensuring the safety and reliability of CNPs. Furthermore, integration of imaging-guided delivery could equip next-generation biomimetic nanoplatforms with enhanced precision and multifunctionality. Collectively, advances in standardized manufacturing and precision delivery are expected to enhance the reproducibility and therapeutic performance of CNPs, thereby accelerating their transition from laboratory research to clinical application for AS.

## Figures and Tables

**Figure 1:**
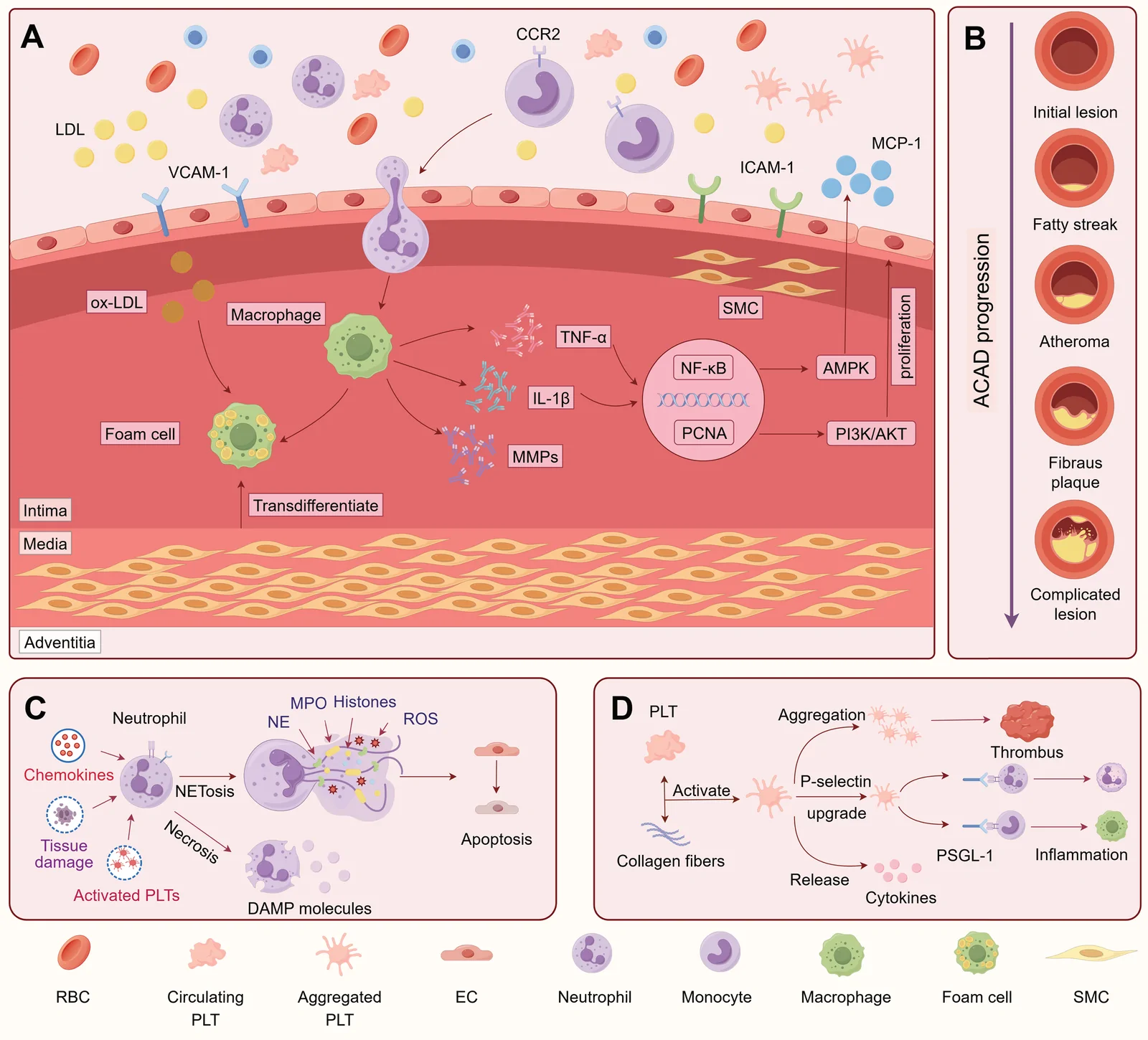
** Schematic illustration of the key pathological processes underlying AS initiation, progression, and complications.** (A) Endothelial dysfunction initiates atherosclerosis by increasing vascular permeability and upregulating adhesion molecules (VCAM-1 and ICAM-1) and MCP-1. CCR2 positive monocytes are recruited into the intima, where they differentiate into macrophages and take up oxidized low-density lipoprotein (ox-LDL), forming foam cells. Activated macrophages promote inflammation and matrix remodeling. Meanwhile, vascular smooth muscle cells (VSMCs) undergo phenotypic switching, migration, and proliferation through pathways such as NF-κB and PI3K/AKT. (B) Atherosclerotic lesions progress from early lesions to advanced plaques, which may cause cardiovascular complications. (C) During plaque progression, activated neutrophils undergo NETosis and release neutrophil extracellular traps (NETs) containing myeloperoxidase (MPO), histones, neutrophil elastase (NE), and reactive oxygen species (ROS), thereby exacerbating vascular inflammation and cell injury. In addition, necrotic cells release damage-associated molecular patterns (DAMPs), further amplifying inflammatory responses. (D) Plaque rupture or endothelial erosion exposes subendothelial collagen, triggering PLT activation and thrombus formation. Subsequently, activated PLTs increase the surface expression of P-selectin, which binds to P-selectin glycoprotein ligand-1 (PSGL-1) on neutrophils and macrophages. This interaction recruits leukocytes and promotes inflammation. Created by figdraw.com.

**Figure 2:**
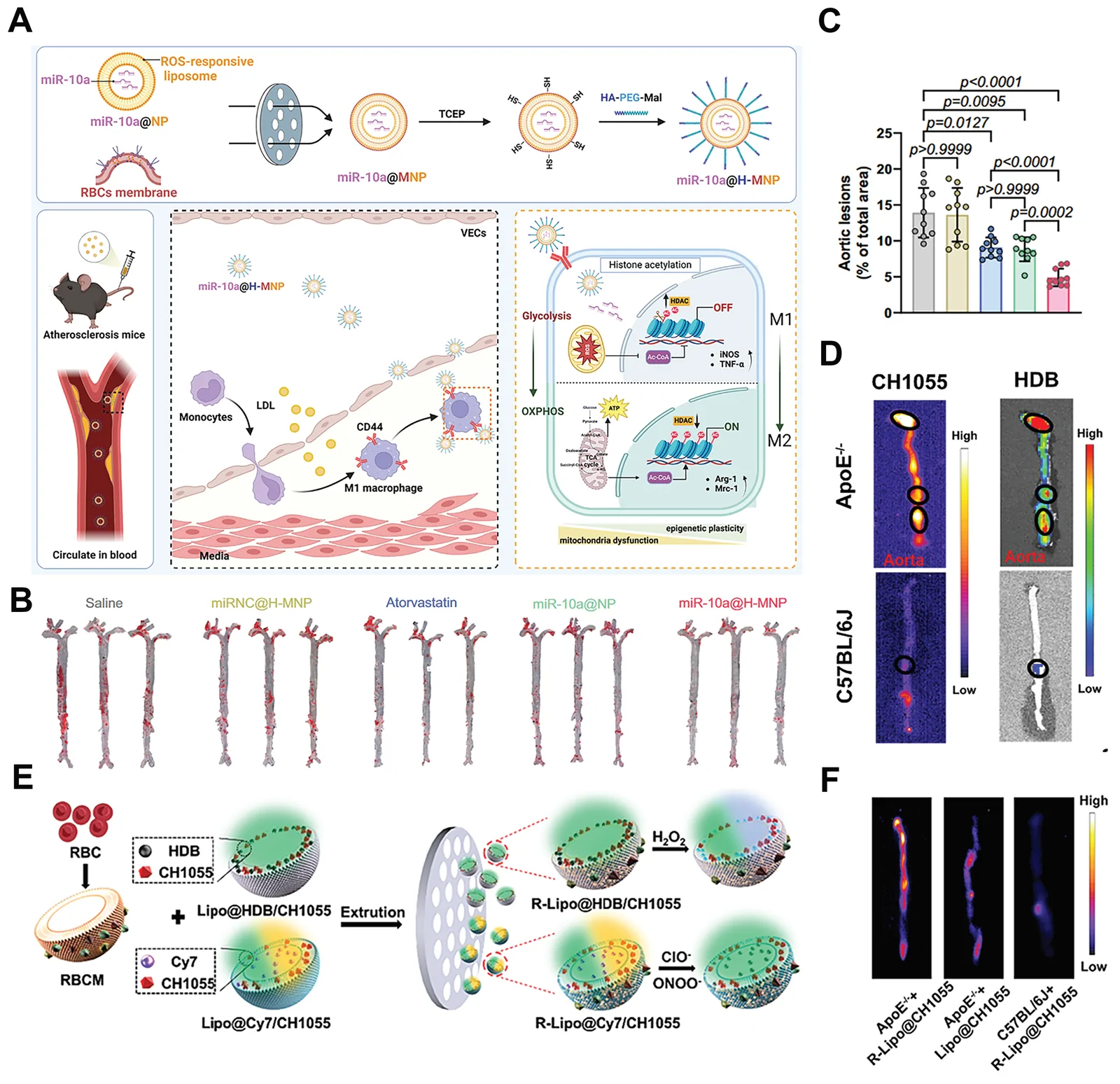
** RBC membrane-derived nanomedicines for AS theranostics.** (A) Schematic illustration of the preparation of miR-10a@H-MNP and its application in the treatment of AS. (B) Representative en face ORO staining images of aortas from *ApoE^-/-^* mice following different treatments. (C) Quantification of the plaque areas shown in (B). Adapted with permission from 87, copyright 2025, Springer Nature. (D) Representative ex vivo fluorescence images of aortas collected 24 h after injection of R-Lipo@HDB/CH1055. (E) Schematic illustration of the preparation of R-Lipo@HDB/CH1055 and R-Lipo@Cy7/CH1055. (F) Representative ex vivo fluorescence images of aortas from different treatment groups. Adapted with permission from 85, copyright 2023, Elsevier B.V. Reproduced with permission of the Licensor through PLSclear. All the data mentioned above were illustrated as mean ± SD. *P < 0.05, **P < 0.01, ***P < 0.001 by one-way ANOVA.

**Figure 3:**
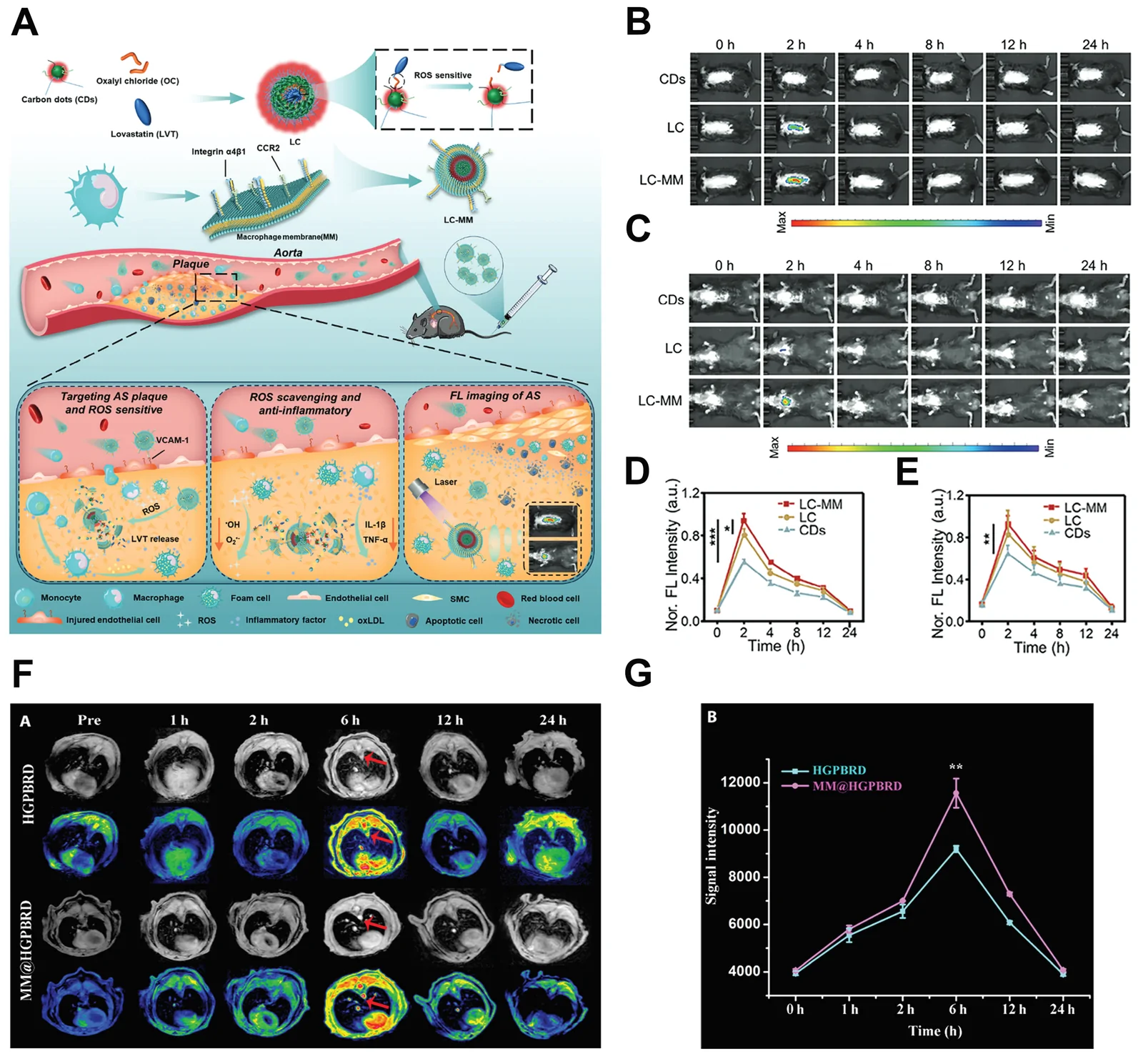
** Macrophage membrane-derived nanoprobes for AS diagnosis.** (A) Schematic illustration of the preparation of LC-MM and its application in AS. (B-C) Representative in vivo fluorescence images of atherosclerotic plaques in *ApoE^-/-^* mice following intravenous injection of CDs, LC, or LC-MM, acquired in the prone (B) and supine (C) positions. (D-E) Quantitative analysis of the fluorescence signal intensities corresponding to (B) and (C), respectively. Adapted with permission from 94, copyright 2024, John Wiley and Sons. (F) Representative T1-weighted MR images of aortic plaques (red arrows) in AS model mice at 1, 2, 6, 12, and 24 h after tail vein injection of MM@HGPBRD or HGPBRD. (G) Quantitative analysis of the MR signal intensities of MM@HGPBRD and HGPBRD. Adapted from 93 under the CC BY license, copyright 2025, AAAS. All the data mentioned above were illustrated as mean ± SD. *P < 0.05, **P < 0.01, ***P < 0.001 by one-way ANOVA.

**Figure 4:**
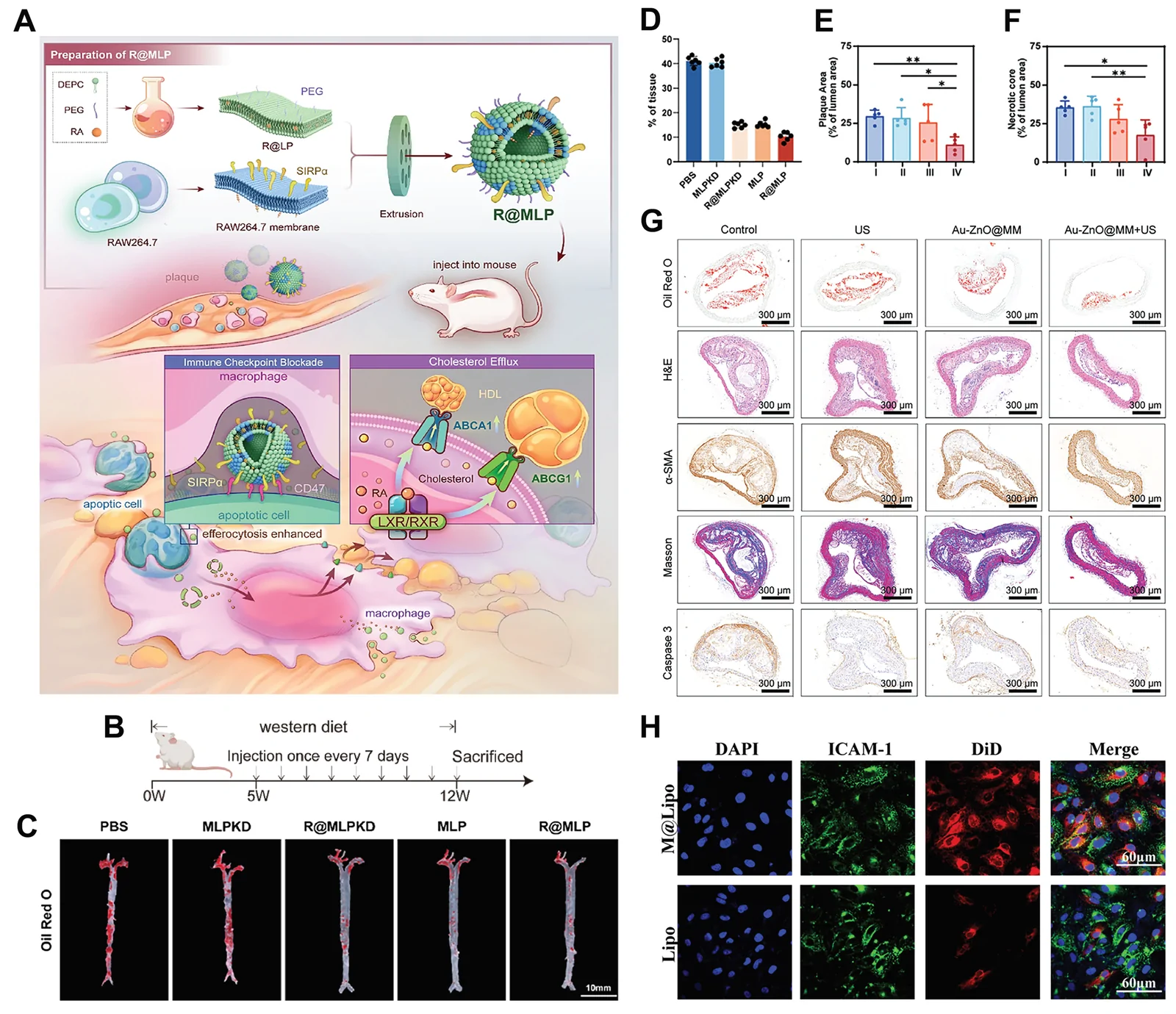
** Macrophage membrane-derived nanomedicines for AS therapy.** (A) Schematic illustration of the fabrication and therapeutic mechanism of R@MLP. (B) Schematic illustration of the in vivo experimental design. (C) Representative en face ORO-stained aortas collected from atherosclerotic mice following different treatments. (D) Quantification of the plaque areas in the aortas after R@MLP treatment. Adapted from 95 under the CC BY license, copyright 2025, Elsevier B.V. (E-F) Quantification of the plaque area-to-lumen ratio (E) and necrotic core-to-lumen ratio (F) after treatment. (G) Representative images of aortic arch sections stained with ORO, H&E, and Masson's trichrome, together with immunohistochemical staining for α-SMA and caspase-3 following treatments. Adapted with permission from 97, copyright 2024, John Wiley and Sons. (H) Representative confocal fluorescence images showing Lipo/M@Lipo (red), HUVECs (blue), and ICAM-1 (green) in LPS-treated cells. Adapted from 96 under the CC BY license, copyright 2023, Springer Nature. All the data mentioned above were illustrated as mean ± SD. *P < 0.05, **P < 0.01, ***P < 0.001 by one-way ANOVA.

**Figure 5:**
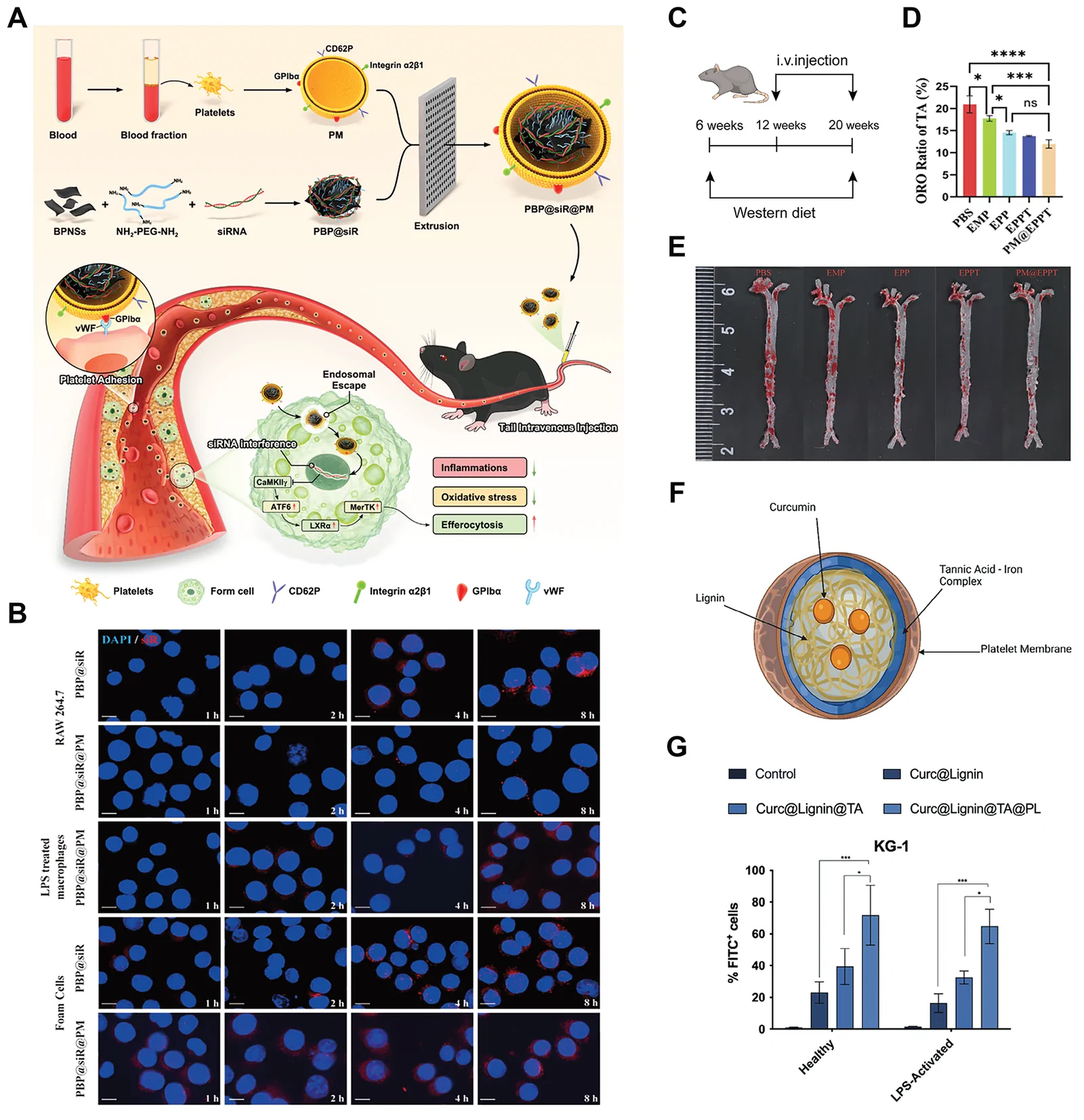
** PLT membrane-derived nanomedicines for AS therapy.** (A) Schematic illustration of PBP@siR@PM for AS therapy. (B) Representative confocal fluorescence images showing the cellular uptake of PBP@siR and PBP@siR@PM by normal macrophages, inflammatory macrophages, and foam cells. Scale bar = 5 μm. Adapted with permission from 107, copyright 2024, Elsevier B.V. Reproduced with permission of the Licensor through PLSclear. (C) Schematic illustration of the in vivo experimental design. (D-E) Representative images of en face ORO-stained aortas from mice treated with PBS, EMP, EPP, EPPT, or PM@EPPT. Adapted from 108 under the CC BY license, copyright 2025, Frontiers Editorial Office. (F) Schematic illustration of Curc@Lignin@TA@PL. (G) Mean fluorescence intensity of CD86 expression in KG-1 cells after 48 h of incubation. Adapted from 109 under the CC BY license, copyright 2024, John Wiley and Sons. All the data mentioned above were illustrated as mean ± SD. *P < 0.05, **P < 0.01, ***P < 0.001 by one-way ANOVA.

**Figure 6:**
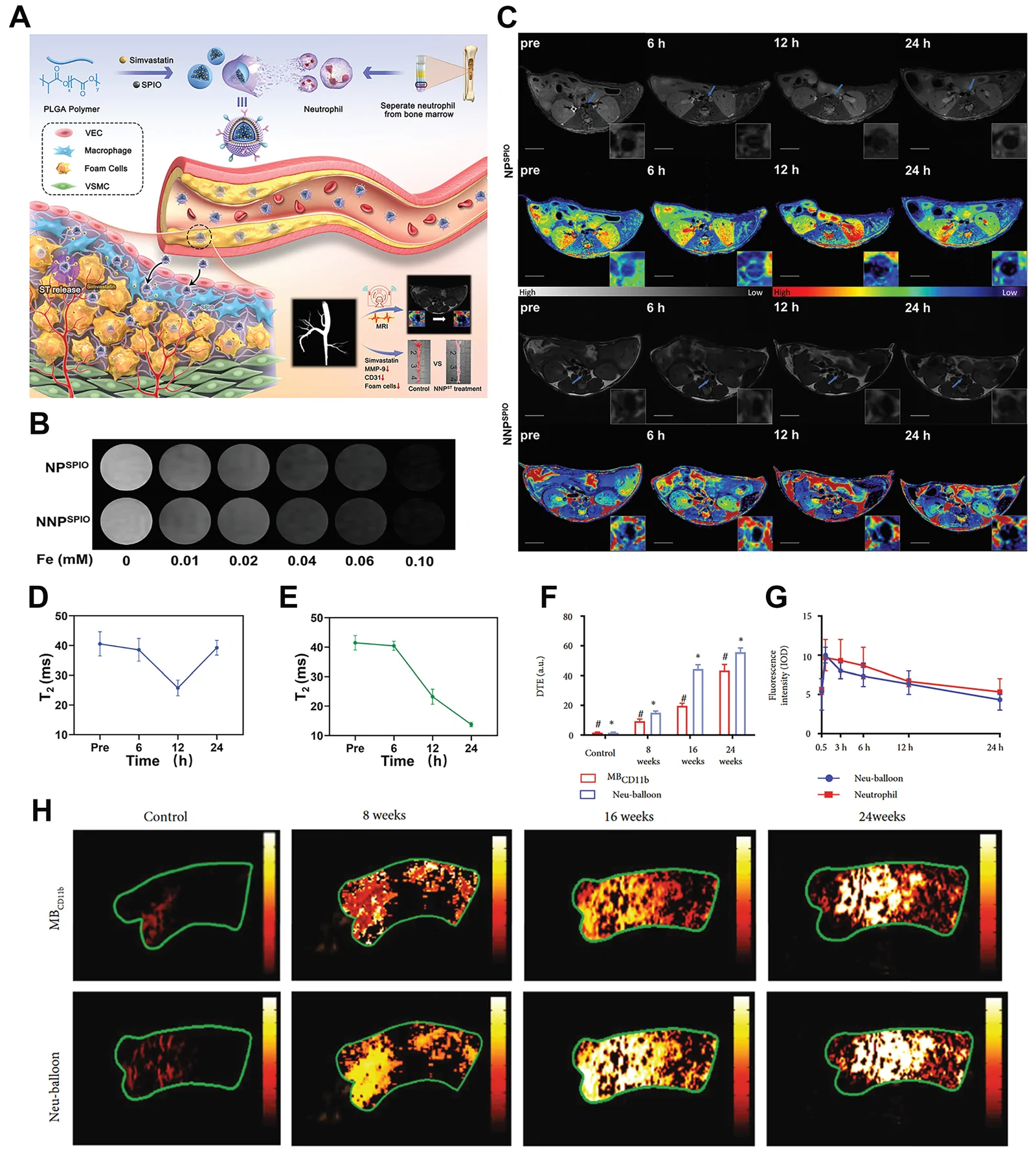
** Neutrophil membrane-derived nanoprobes for AS diagnosis.** (A) Schematic illustration of the synthesis of NNP^ST^ and its application in AS. (B) Representative ex-vivo T_2_-weighted MR images of NP^SPIO^ and NNP^SPIO^ at different Fe concentrations. (C) Representative T_2_-weighted MR images and corresponding pseudocolor images of *ApoE^-/-^* mice acquired before and at different time points after injection of NP^SPIO^ or NNP^SPIO^. Scale bar = 5 mm. (D-E) Quantification of the T_2_ relaxation times of atherosclerotic plaques following injection of NP^SPIO^ (D) and NNP^SPIO^ (E). Adapted from 115 under the CC BY license, copyright 2024, Elsevier. (F) Quantification of the PA signal intensity shown in (H). (G) Quantification of fluorescence intensity within atherosclerotic plaques after probe delivery by Neu-balloons or free neutrophils. (H) Representative PA signals in the Neu-balloon and MB_CD11b_-treated groups at 8, 16, and 24 weeks. Adapted from 116 under the CC BY license, copyright 2022, AAAS. All the data mentioned above were illustrated as mean ± SD. *P < 0.05, **P < 0.01, ***P < 0.001 by one-way ANOVA.

**Figure 7:**
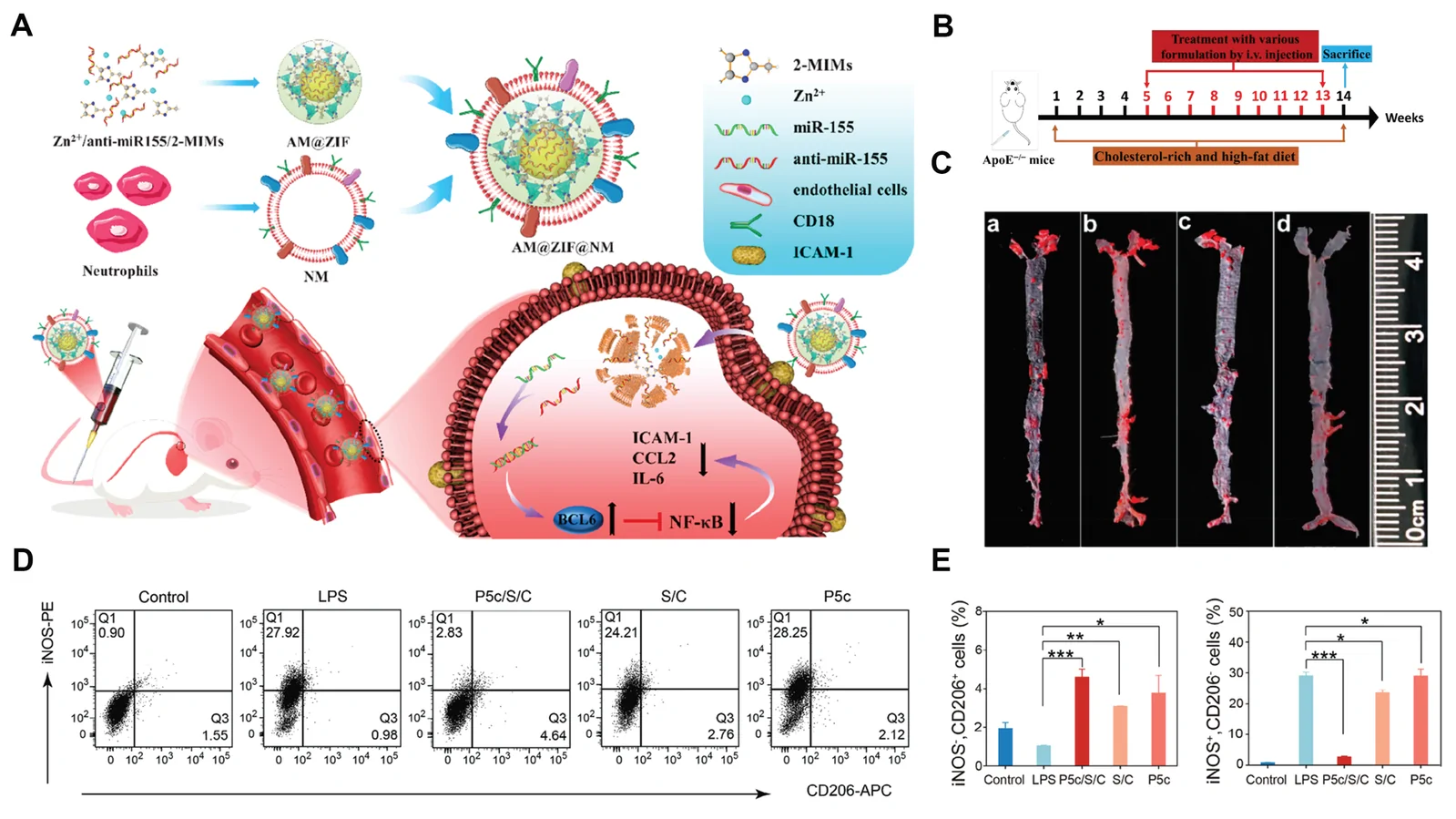
** Neutrophil membrane-derived nanomedicines for AS therapy.** (A) Schematic illustration of the biomimetic AM@ZIF@NM NPs for targeted AS therapy. (B) Schematic illustration of the therapeutic protocol for evaluating AM@ZIF@NM NPs in *ApoE^-/-^* mice. (C) Representative images of en face ORO-stained aortas from mice treated with different formulations. Adapted with permission from 119, copyright 2023, American Chemical Society. (D) Representative flow cytometry analysis of macrophage subsets in LPS-stimulated RAW264.7 macrophages following 6 h incubation with different formulations. (E) Percentages of M2 (iNOS^-^ and CD206^+^) and M1 (iNOS^+^ and CD206^-^) macrophages shown in (D). Adapted with permission from 117, copyright 2024, John Wiley and Sons. All the data mentioned above were illustrated as mean ± SD. *P < 0.05, **P < 0.01, ***P < 0.001 by one-way ANOVA.

**Figure 8:**
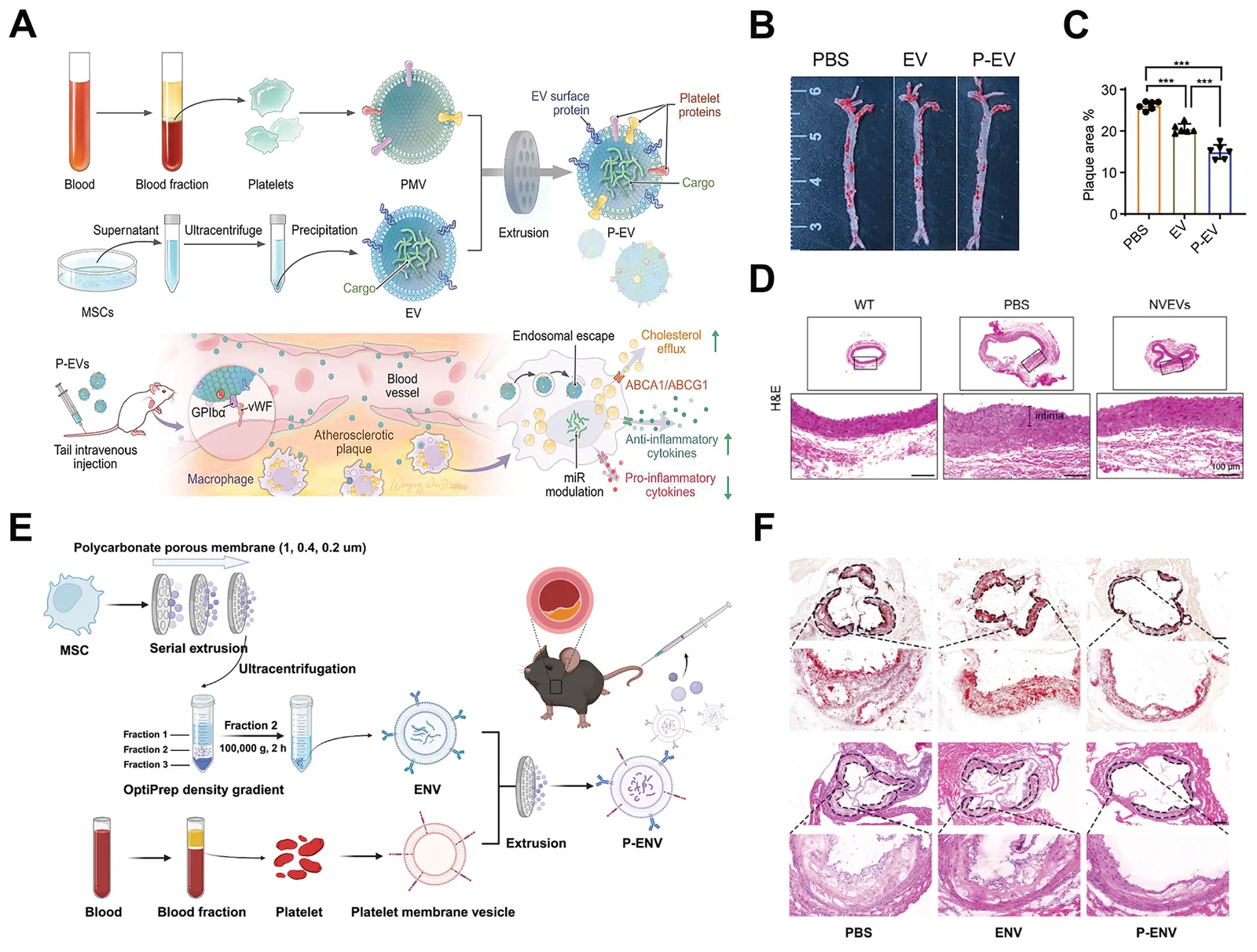
** EV-derived nanomedicines for AS therapy.** (A) Schematic illustration of the fabrication and mechanism of P-EVs for the treatment of AS. (B) Representative en face ORO-stained aortas from mice treated with PBS, EVs, or P-EVs. (C) Quantification of the plaque area in the aortas. Adapted with permission from 131, copyright 2023, Elsevier B.V. Reproduced with permission of the Licensor through PLSclear. (D) Representative H&E-stained sections of balloon-injured arteries showing vascular morphology. Adapted with permission from 132, copyright 2025, John Wiley and Sons. (E) Schematic illustration of the synthesis of P-ENVs for AS therapy. (F) Representative ORO- and H&E-stained images of aortic roots from mice treated with PBS, ENVs, or P-ENVs. Scale bar = 200 μm. Adapted with permission from 133, copyright 2024, Informa UK Limited. Reproduced with permission of the Licensor through PLSclear. All the data mentioned above were illustrated as mean ± SD. *P < 0.05, **P < 0.01, ***P < 0.001 by one-way ANOVA.

**Table 1:** Summary of the main biomimetic nanomedicines for AS diagnosis

Biomimetic Source	Nanomedicines	Particle Size	Targeting Mechanism	Animal Model	Imaging Modality	Reference
Macrophages	MφMB-Au	50 nm	Inflammatory chemotaxis	*ApoE^-/-^* mouse	US/PA	75
RBC Membrane	R-Lipo@HDB/CH1055 R-Lipo@Cy7/CH1055	136.3 nm/147.6 nm	Passive targeting	*ApoE^-/^*^-^ mouse	FI	85
Macrophage Membrane	MM@HGPBRD	125.5 nm	Inflammatory chemotaxis	*ApoE^-/-^* mouse	MRI	93
Macrophage Membrane	LC-MM	35 nm	Integrin α4β1/VCAM-1 recognition	*ApoE^-/-^* mouse	FI	94
PLT Membrane	PM@CBNP	128.1 nm	GP Ib-IX complex/vWF binding	*ApoE^-/-^* mouse	FI	101
PLT Membrane	PM/Fe_3_O_4_@PLGA	280.33 nm	GP Ib-IX complex/vWF binding	*ApoE^-/-^* mouse	MRI	102
PLT Membrane	PGA@PMP	185.1 nm	ICAM-1/VCAM-1 recognition	*ApoE^-/-^* mouse	PA	105
PLT Membrane	RBT3-NO-PEG@PM	130 nm	Glycoprotein receptors interaction	*ApoE^-/-^* mouse	FI	106
Neutrophil Membrane	NNP^ST^	116.8 nm	LFA-1/ PSGL-1/ TLR4	*ApoE^-/-^* mouse	MRI	115
Neutrophil Membrane	Neu-Balloons	8.4 ± 1.2 μm	CD11b/ ICAM-1/ E-selectin	*ApoE^-/-^* mouse	FI	116

**Table 2:** Summary of the key biomimetic nanomedicines for AS treatment

Biomimetic Source	Nanomedicines	Particle Size	Mechanisms	Animal Model	Plaque Reduction Percentages	Reference
Neutrophil	MNP_pep	23.9 nm	ROS ↓	*ApoE^-/-^* mouse	39.5%	76
RBC Membrane	Nanoghost	263.0 nm	M2 polarization ↑	Rabbit	-	86
RBC Membrane	miR-10a@H-MNP	145.0 nm	M2 polarization ↑	*ApoE^-/-^* mouse	64.0%	87
RBC Membrane	RBC@P-LVTNPs	254.0 nm	Lipid deposition ↓	*ApoE^-/-^* mouse	68.5%	88
Macrophage Membrane	R@MLP	116.6 nm	Cholesterol efflux ↑ Macrophage phagocytosis ↑	*ApoE^-/-^* mouse	69.5%	95
Macrophage Membrane	(Lipo+M)@E NPs	183.2 nm	PCSK9 level ↓	*ApoE^-/-^* mouse	81.2%	96
Macrophage Membrane	Au-ZnO@MM	124.8 nm	Cholesterol efflux↑ Macrophage phagocytosis ↑	*ApoE^-/-^* mouse	58.0%	97
PLT Membrane	PBP@siR@PM	140.0 nm	ROS ↓ Macrophage phagocytosis ↑	*ApoE^-/-^* mouse	82.3%	107
PLT Membrane	PM@EPPT	105.3 nm	ROS ↓ Mitophagy ↑	*ApoE^-/-^* mouse	42.8%	108
PLT Membrane	Curc@Lignin@TA@PL	418 nm	M2 polarization ↑	-	-	109
Neutrophil Membrane	NeuM@P5c/S/C	316.9 nm	M2 polarization ↑ Macrophage autophagy ↑	*ApoE^-/-^* mouse	74.3%	117
Neutrophil Membrane	Ptdser-NM-Lipo/Fer-1	174.9 nm	ROS ↓	*ApoE^-/-^* mouse	-	118
Neutrophil Membrane	AM@ZIF@NM	112.5 nm	MicroRNA-155 level ↓	*ApoE^-/-^* mouse	72.9%	119
EVs	P-EVs	129.3 nm	ROS ↓ Lipid deposition ↓	*ApoE^-/-^* mouse	42.8%	131
EVs	NVEVs	97.6 nm	Cholesterol efflux ↑	SD Rats	-	132
EVs	P-ENVs	150.9 nm	Cholesterol efflux ↑	*ApoE^-/-^* mouse	72.0%	133

**Table 3:** Targeting strategies based on disease stage progression

Disease stage	Key pathological features	Dominant targets	Therapeutic goal	Biomimetic design strategy	Representative biomimetic materials
Early lesion	Endothelial dysfunction, Increased vascular permeability, Mild inflammation	Activated endothelium	Restore endothelial function	Endothelium-targeted biomimetic delivery	Neutrophil membrane-coated NPs
Progressive lesion	Lipid accumulation, Chronic inflammation, Immune cells recruitment	Inflammatory plaque	Modulate inflammation and lipid metabolism	Immune cell-mimetic targeting to inflamed regions	Macrophage membrane-coated NPs; Neutrophil membrane-coated NPs
Advanced lesion	Necrotic core formation, ECM remodeling, Oxidative stress	Oxidative stress-rich plaque	Stabilize plaque microenvironment	Stimuli-responsive biomimetic nanomedicines	Macrophage membrane-coated NPs;
Thrombotic lesion	Thrombus formation, Vascular occlusion	Thrombus	Target thrombus and Prevent occlusion	Thrombus-targeting drugs with high affinity to clot components	PLT membrane-coated NPs

**Table 4:** Targeting strategies based on plaque phenotype

Plaque phenotype	Pathological features	Dominant targets	Therapeutic goal	Biomimetic design strategy	Representative biomimetic materials
Vulnerable plaque	Thin fibrous cap, Large necrotic core, Intense inflammation, High rupture risk	MMPs, ROS, Inflammatory cytokines, Activated macrophages	Suppress inflammation, Inhibit ECM degradation, Promote plaque stabilization	Target inflamed endothelium and necrotic core, Cytokine sequestration	Macrophage membrane-coated NPs, Neutrophil membrane-coated NPs
Stable plaque	Thick fibrous cap, Small lipid core, Low inflammation, Lower rupture risk	Low-level inflammatory mediators	Maintain vascular homeostasis	Long circulation, Sustained drug delivery	RBC membrane-coated NPs, EVs

**Table 5:** Recent patent applications on biomimetic nanomedicines for AS

Biomimetic Source	Patent Number	Applicant/Assignee	Publication Year
RBC membrane	CN121695107A	Qingdao University	2026
RBC-macrophage hybrid membrane	CN119792573A	Ningxia Medical University	2025
Macrophage membrane	WO2026060590A1	Guangzhou Medical University	2026
Macrophage membrane	CN121489903A	Guangzhou University of Chinese Medicine	2026
Macrophage membrane	CN120037392A	Chongqing Medical University	2026
PLT membrane	CN118892467A	Huazhong University of Science and Technology	2024
PLT membrane	CN121243154A	Ningxia Medical University	2026
Exosomes	CN115590882A	Central South University	2023
EVs	CN121343873A	Dalian University of Technology	2026
EVs	CN121648319A	Institute of Biomedical Engineering, Chinese Academy of Medical Sciences	2026

## Abbreviations

AS: Atherosclerosis
CAD: Coronary Artery Disease
NP: Nanoparticle
CM-CNPs: cell membrane–coated nanoparticles
CT: Computed Tomography
MRI: Magnetic Resonance Imaging
LDL: Low-Density Lipoprotein
PCSK9: Proprotein Convertase Subtilisin/Kexin Type 9
EVs: Extracellular Vesicles
ECs: Endothelial Cells
MCP-1: Monocyte Chemoattractant Protein-1
IL-8: Interleukin-8
VCAM-1: Vascular Cell Adhesion Molecule-1
ICAM-1: Intercellular Adhesion Molecule-1
ROS: Reactive Oxygen Species
ox-LDL: oxidized Low-Density Lipoprotein
CCR2: C–C Chemokine Receptor Type 2
TNF-α: Tumor Necrosis Factor-Alpha
IL-1β: Interleukin-1 Beta
PLTs: Platelets
GPVI: Glycoprotein VI
PSGL-1: P-Selectin Glycoprotein Ligand-1
NETs: Neutrophil Extracellular Traps
VSMCs: Vascular Smooth Muscle Cells
ECM: Extracellular Matrix
MMPs: Matrix Metalloproteinases
RBCs: Red Blood Cells
IPH: Intraplaque Hemorrhage
DAMPs: Damage-Associated Molecular Patterns
US: Ultrasound
PA: Photoacoustic
FI: Fluorescence Imaging
RNAi: RNA Interference
miRNA: microRNA
siRNA: small interfering RNA
MSC: Mesenchymal Stem/Stromal Cell
SD: Standard Deviation
ASO: Antisense Oligonucleotide
NF-κB: Nuclear Factor Kappa-Light-Chain-Enhancer of Activated B Cells
LDLR: Low-Density Lipoprotein Receptor
eLDL: Electronegative Low-Density Lipoprotein
BVOs: Blood Vessel Organoids
AI: Artificial Intelligence
